# Ferritin Is Required in Multiple Tissues during *Drosophila melanogaster* Development

**DOI:** 10.1371/journal.pone.0133499

**Published:** 2015-07-20

**Authors:** Nicanor González-Morales, Miguel Ángel Mendoza-Ortíz, Liisa M. Blowes, Fanis Missirlis, Juan R. Riesgo-Escovar

**Affiliations:** Departamento de Neurobiología del Desarrollo y Neurofisiología, Instituto de Neurobiología, Universidad Nacional Autónoma de México, Campus UNAM Juriquilla, Querétaro, 76230, México; Alexander Fleming Biomedical Sciences Research Center, GREECE

## Abstract

In *Drosophila melanogaster*, iron is stored in the cellular endomembrane system inside a protein cage formed by 24 ferritin subunits of two types (Fer1HCH and Fer2LCH) in a 1:1 stoichiometry. In larvae, ferritin accumulates in the midgut, hemolymph, garland, pericardial cells and in the nervous system. Here we present analyses of embryonic phenotypes for mutations in *Fer1HCH*, *Fer2LCH* and in both genes simultaneously. Mutations in either gene or deletion of both genes results in a similar set of cuticular embryonic phenotypes, ranging from non-deposition of cuticle to defects associated with germ band retraction, dorsal closure and head involution. A fraction of ferritin mutants have embryonic nervous systems with ventral nerve cord disruptions, misguided axonal projections and brain malformations. Ferritin mutants die with ectopic apoptotic events. Furthermore, we show that ferritin maternal contribution, which varies reflecting the mother’s iron stores, is used in early development. We also evaluated phenotypes arising from the blockage of COPII transport from the endoplasmic reticulum to the Golgi apparatus, feeding the secretory pathway, plus analysis of ectopically expressed and fluorescently marked Fer1HCH and Fer2LCH. Overall, our results are consistent with insect ferritin combining three functions: iron storage, intercellular iron transport, and protection from iron-induced oxidative stress. These functions are required in multiple tissues during *Drosophila* embryonic development.

## Introduction

Iron is the most abundant transition metal on earth, commonly found at active sites of enzymes in the form of heme or iron-sulfur clusters, or as mono-nuclear or di-nuclear iron [1]. Because of the high reactivity between iron and oxygen, iron has become a key player in aerobic metabolism. Excessive iron catalyzes noxious oxidative stress. Therefore, iron concentration within subcellular compartments and extracellular fluids is tightly regulated. The Divalent Metal Transporter 1 (DMT1) is responsible for cellular iron uptake [2,3].

Ferritin participates in iron homeostasis as the main iron storage complex in both prokaryotes and eukaryotes [4,5]. Ferritin major form (holoferritin) is cytosolic and consists of 24 H and L protein chains subunits that assembles into a cage-like structure. This complex sequesters up to 4,500 iron atoms in its interior. The H chains contain a ferroxidase center necessary for iron internalization while the L chains contain acidic groups exposed in the interior surface of the holoferritin complex facilitating iron mineralization [6]. Ferritin genes are regulated during translation by the iron regulatory protein (IRP) binding to an iron responsive element (IRE) in the ferritins mRNAs 5' untranslated region [7–9]. The discovery of ferritin receptors Scara5 [10] and Tim2 [11] in mice has led to the idea that ferritin might be involved in iron transport [12,13]; however, this idea remains controversial [14].

In insects, ferritin shells have an H12L12 organization due to inter- and intra- subunit disulfide-bonds that ensure protein folding and assembly [15]. Intracellular ferritin in most insects is directed to the endoplasmic reticulum and the Golgi apparatus [16,17]. The *Drosophila melanogaster* genome encodes three ferritin genes: *Ferritin 1 heavy chain homologue (Fer1HCH*) and *Ferritin 2 light chain homologue (Fer2LCH)* that together produce the major ferritin complex [18–20]. *Ferritin 3 heavy chain homologue (Fer3HCH)* encodes the mitochondrial ferritin, predominantly expressed in testis [16,21].

The *Drosophila* Iron Regulatory Protein-1A (IRP-1A), in its iron-sulfur cluster depleted modality, binds IREs thereby regulating the translation of a subset of *Fer1HCH* mRNA transcripts [22]. The sole *Drosophila* DMT1 homolog [23] was originally isolated as a gustatory mutant named *Malvolio (Mvl)* [24,25].

Previous work has shown that ferritin is required for embryonic and larval development[17,26] and that the ferroxidase center of the H chain is essential [17], but the specific phenotypes of the ferritin mutants have not been studied. It has been proposed that *Drosophila* ferritin might also serve a role as an extracellular source of iron [10,26–28], but functional analyses supporting this exist only for the secreted ferritin of ticks [29,30].

Here we analyze *Fer1HCH* and *Fer2LCH* mutant embryonic phenotypes. We show that key functions of the ferritin subunits are likely mediated through the ferritin complex, as single mutant phenotypes are indistinguishable from double mutants during embryogenesis. We also show that ferritin mutant phenotypes can be enhanced when embryos have reduced or are deprived of ferritin maternal contribution, by limiting iron uptake in parental diets, or by induction of germ line clones, respectively. Ferritin mutant phenotypes encompass a wide range of cuticular phenotypes, implying widespread embryonic functions. A strong central nervous system (CNS) defect results from mutations in ferritin genes. We show that this phenotype is accompanied by ectopic apoptosis. Last, we show that blocking the intracellular secretory pathway during embryogenesis results in the mislocalisation of ferritin, and that ectopically expressed and marked ferritin subunits are in tissues and organs where they were not synthesized, also implying transport. We hypothesize that failure of ferritin transport contributes to mutant phenotypes and lethality.

## Results

### Pleiotropic phenotypes of ferritin mutants in embryonic cuticle preparations

Nüsslein-Volhard and Wieschaus examined *Drosophila* embryonic cuticular defects extensively (affecting in principle the ectodermally derived tegument and cuticle deposition) and showed it to be a useful and comprehensive way of analyzing genes required during embryogenesis, since many times mutations affecting the cuticle also affect other embryonic tissues and cell types [31]. We analyzed cuticles of previously described transposon-induced loss-of-function ferritin alleles: *Fer1HCH451* and *Fer2LCH35* [17,26]. We also used and compared them with a null mutation for both ferritin genes *Df(3R)Fer* [32], a GFP-trap allele, *Fer1HCHG188* [17], and a new allele, *Fer2LCHΔ17*, that fails to complement *Fer1HCH451*, *Fer1HCHG188*, and *Fer2LCH35* (see Materials and Methods). All of these alleles are lethal.

**Fig 1:**
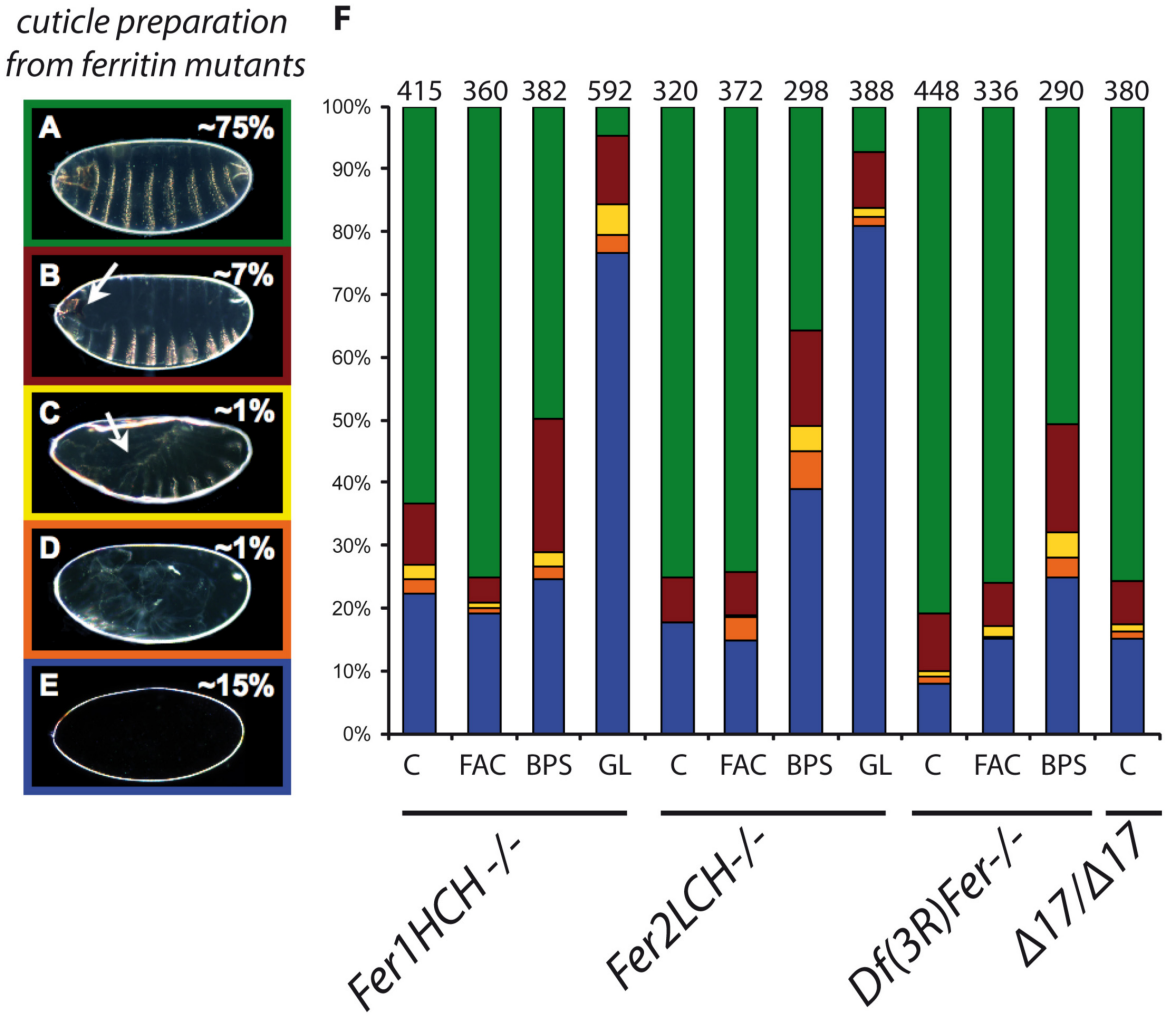
Ferritin mutants result in a variety of cuticle phenotypes quantified by different colors. Examples are shown of (A) wild type cuticle (green), (B) head involution defects (red), (C) dorsal closure defects (yellow), (D) germ band retraction defects (orange), and (E) no cuticle deposition (blue). (F) Percentages of cuticular phenotypes of ferritin mutants. An enhancement of the earlier phenotypes was seen in mutant embryos whose mothers were fed BPS, which was dramatic in embryos derived from ferritin mutant germline clones. C: normal diet, FAC: high iron diet, BPS: low iron diet, GL: germline clones, n: number of embryos examined per genotype. An asterisk denotes statistical difference compared to the control lane at p<0.001.

A significant number of ferritin mutant embryos die during embryogenesis (36% for *Fer2LCH35*, 43% for *Fer1HCH451*, and 90% for *Df(3R)Fer*; in our hands in these experiments in control flies only 1% of embryos die during embryogenesis. Differing numbers of mutant cuticles may just reflect level of lack-of-function attained in the different mutant genotypes, as opposed to qualitative differences, since they all exhibit exactly the same mutant phenotypes). Cuticle preparations of zygotic ferritin homozygous mutants are mostly wildtype (approximately 75%), but a fraction (overall 25%) has no cuticle deposition, or cuticular defects (a small fraction have germ band extension and retraction, dorsal closure, and head involution defects, among others; Fig 1). Quantification showed that the majority of mutant embryos presented either a normal cuticle (~75%) or no cuticle at all (~15%). A significant finding was that the ferritin mutant genotypes tested showed similar percentages of mutant phenotypes. We interpret this to mean that individual ferritin subunits only function in concert (as the ferritin complex, holoferritin) and do not have vital subunit-specific functions. These diverse phenotypes also suggest that ferritin is required in several tissues during development, consistent with differential iron needs in cells and tissues. Notably, a number of iron sulfur cluster proteins are induced during the final stages of embryogenesis to support glycolysis in an aerobic environment [33]. As cuticle deposition occurs during the last stages of embryonic development, defects in cuticle deposition may have a late cause. On the other hand, mutant cuticles pinpoint defects manifested earlier during development: germband extension-retraction (stages 9–12), dorsal closure (stages 13–15), and head involution (stages 14–16). Overall, we found defects from mid-development (stage 9) onwards. All these defects were not found in control stocks.

### Ferritin maternal contribution is utilized during early embryonic development

Ferritin is maternally contributed [17]. We therefore wondered if our analysis of zygotic mutants would miss early ferritin requirements (even obligate maternally-encoded ferritin requirements) fulfilled by this maternal contribution. To analyze how maternal ferritin functioned during embryogenesis we followed two strategies: a) curtailed iron availability in mothers, where some maternally deposited ferritin mRNAs and proteins would be available in the egg, but iron would be limited, and b) generating female germ line clones without wild type copies of the two main ferritin genes.

A way of reducing ferritin expression and iron availability in adults is to add an iron-specific chelator in the diet (S1 Fig)[16,34]. We hypothesized that reduced overall iron levels would result in decreased ferritin (and iron) maternal contribution and a more severe embryonic phenotype. Homozygous mutant embryos derived from heterozygous adults fed with 200 μM Bathophenanthroline Sulfate (BPS) showed, in general, a doubling of incidence of embryonic defects (particularly head involution), including the no cuticle phenotype (from ~15% to ~30%). We interpret this accruement of cuticle phenotypes, like no cuticle, as due to early embryonic death, sometimes before epidermal differentiation, although we have not excluded a particular requirement for iron in the differentiation of the epidermis, which could provide an alternative explanation for the same last phenotype. The other embryonic phenotypes also became more frequent: U-shaped embryos, indicative of a failure of germ band retraction, augmented from ~2% to ~4%, and embryos failing to complete dorsal closure increased from ~1% to ~4% (Fig 1F; BPS columns). At the same time, the percentage of “wild type” cuticles decreased. Using cuticle preparations, this evidence points to iron levels being critical for all ferritin embryonic functions, and BPS treatment as being a way of generating more extreme lack-of-function conditions. No new mutant phenotypes, different from non-iron reduced zygotic mutants, were detected in these embryos, supporting that ferritin and iron deficits are phenotypically coincidental. Once again, the changes in phenotypic classes abundances were consistently similar in *Fer1HCH451*, *Fer2LCH35*, and *Df(3R)Fer*.

On the other hand, feeding extra iron to adults did not result in a rescue of the zygotic embryonic phenotype of the mutant offspring (Fig 1F, FAC), even though total levels of ferritin in mothers increased (S1 Fig). This shows that maternal ferritin contribution is not sufficient to fully rescue embryogenesis in ferritin homozygous mutants, implying an obligate role for zygotically expressed ferritin in embryonic development.

To further explore whether maternal ferritin only partially rescues zygotic ferritin mutants, especially during early development, we generated homozygous ferritin mutant germ line clones. Most germ line clones had no cuticle or bore cuticles with defects (95% of mutant embryos). The ‘no cuticle’ phenotype percentage changed spectacularly from ~15% to ~80% (Fig 1F). Again, no new mutant phenotypes were seen. This augmentation of phentoypes is in agreement with the BPS experiment above, consistent with early ferritin/iron requirements during embryogenesis. A wild type copy of zygotic ferritin can rescue a small fraction of mutant embryos that were maternally deprived of ferritin, implying no obligate function for maternally supplied ferritin.

Heterozygous embryos without maternal contribution develop normally into adults without defects. Taken together, these results show that maternally supplied ferritin is employed early during development, but can be functionally replaced by zygotically expressed ferritin. We hypothesize that this rescue is due to zygotic ferritin genes being overexpressed in order to compensate for lack of maternal ferritin, sufficient to handle iron needs during embryonic development. It still remains to be seen how iron is maternally deployed in the oocyte when there is no maternal Fer1HCH and Fer2LCH available, as ferritin complex formed from these two proteins is the main iron storage complex in the fly.

The diversity of cuticular phenotypes, and their increase in conditions where iron availability is reduced and/or ferritin is deprived, leads to a common ‘syndrome’ of mutant cuticles, where lack of cuticle deposition becomes particularly abundant. The different mutant embryonic cuticular phenotypes, including their increase in maternally reduced or deprived embryos, are consistent with ferritin requirements several times during development.

### Central Nervous System (CNS) phenotypes of ferritin mutants

The majority of zygotic ferritin mutant embryos derived from heterozygous mothers die with an apparently normal cuticle. Since not all functional requirements in embryonic lethal mutations are reflected as cuticular defects, we also sought to study other tissues and organs. We sought to study an internal organ or tissue that might be affected by lack of ferritin, and contribute to the death of the mutant embryos, an organ or tissue whose defects which might not necessarily reflect in the cuticle. Since ferritin transcripts are first detected in the developing neuroectoderm at stages 7–8 and the protein becomes concentrated in the CNS of first instar larvae [21,35], we asked whether the CNS developed normally in ferritin mutants.

**Fig 2:**
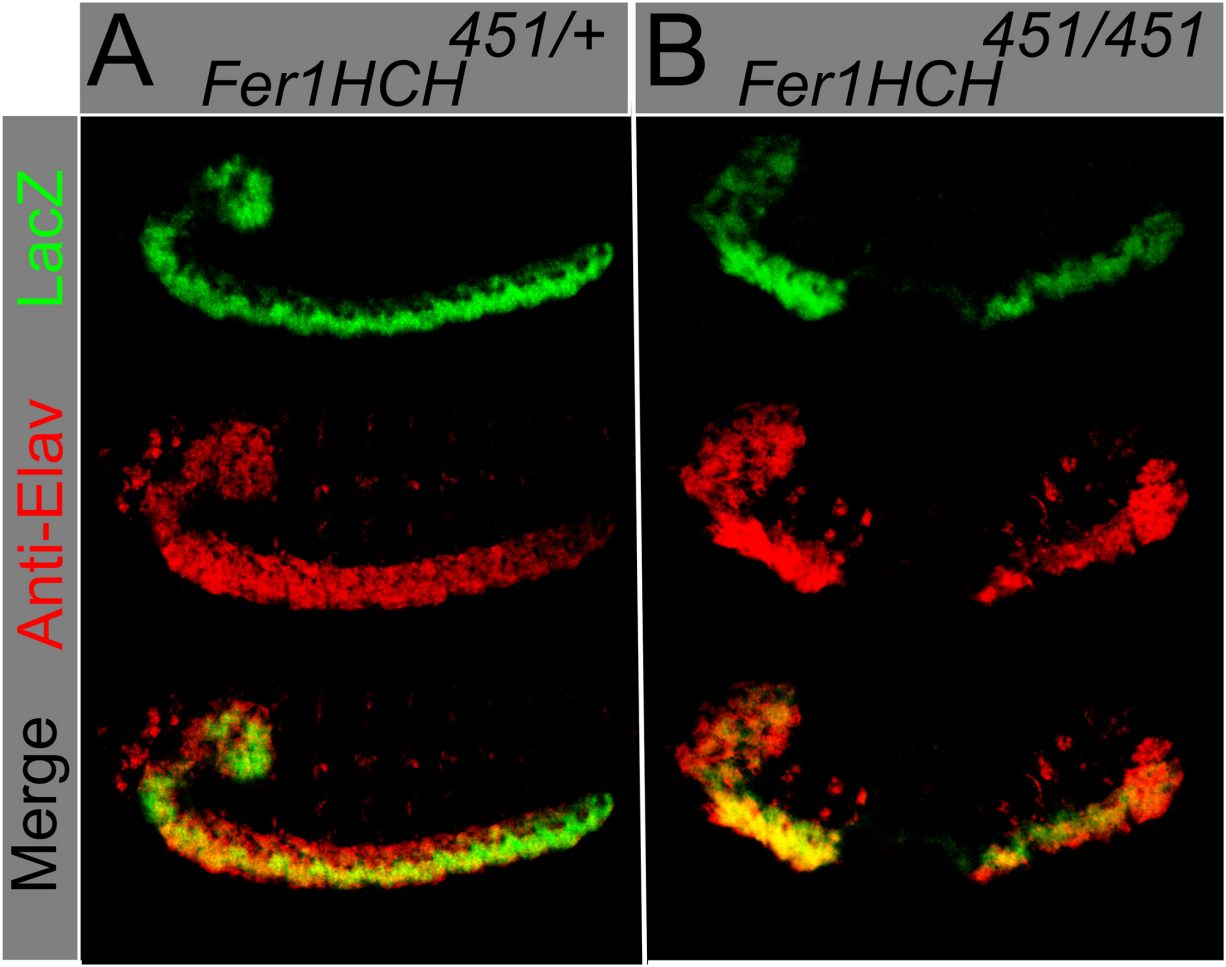
*Fer1HCH^451^ lacZ* enhancer trap is expressed in the embryonic CNS. Using an antibody against Anti- Beta-Galactosidase (green) and an antibody against the neuronal marker Elav (red), colocalization is observed in (A) heterozygous *Fer1HCH^451/+^* and (B) homozygous *Fer1HCH^451^* embryos. Ferritin homozygous mutant embryo has abnormally shaped and separated nervous system.

*Fer1HCH451* carries a *LacZ* element that serves as a reporter for *Fer1HCH* expression. Previous reports have shown that both ferritin subunit transcripts are mainly present in the neuroectoderm, the fat body, and in the midgut in the final stages of embryonic development [16,21,35]. We tested whether this LacZ reporter recapitulates known *Fer1HCH* expression. For this, we monitored *LacZ* activity (due to the reporter transgene) in the anterior midgut upon iron feeding of larvae (a condition that augments endogenous *Fer1HCH* expression; S2 Fig). Since we observed iron-dependent induction of expression, we reasoned that the reporter responds to iron (like the endogenous *Fer1HCH*), and used this *Fer1HCH451* LacZ staining as an enhancer trap to monitor *Fer1HCH* expression in the embryo. *Fer1HCH451*-LacZ is strongly expressed in the neuroectoderm (Fig 2). Co-localization between the neuronal marker Elav and β-galactosidase was also observed in homozygous mutant embryos that have a disrupted CNS (Fig 2). The earliest stage we see defects with Elav antibody are stage 11–12, some time after nervous system ferritin expression is seen at stages 7–8.

**Fig 3:**
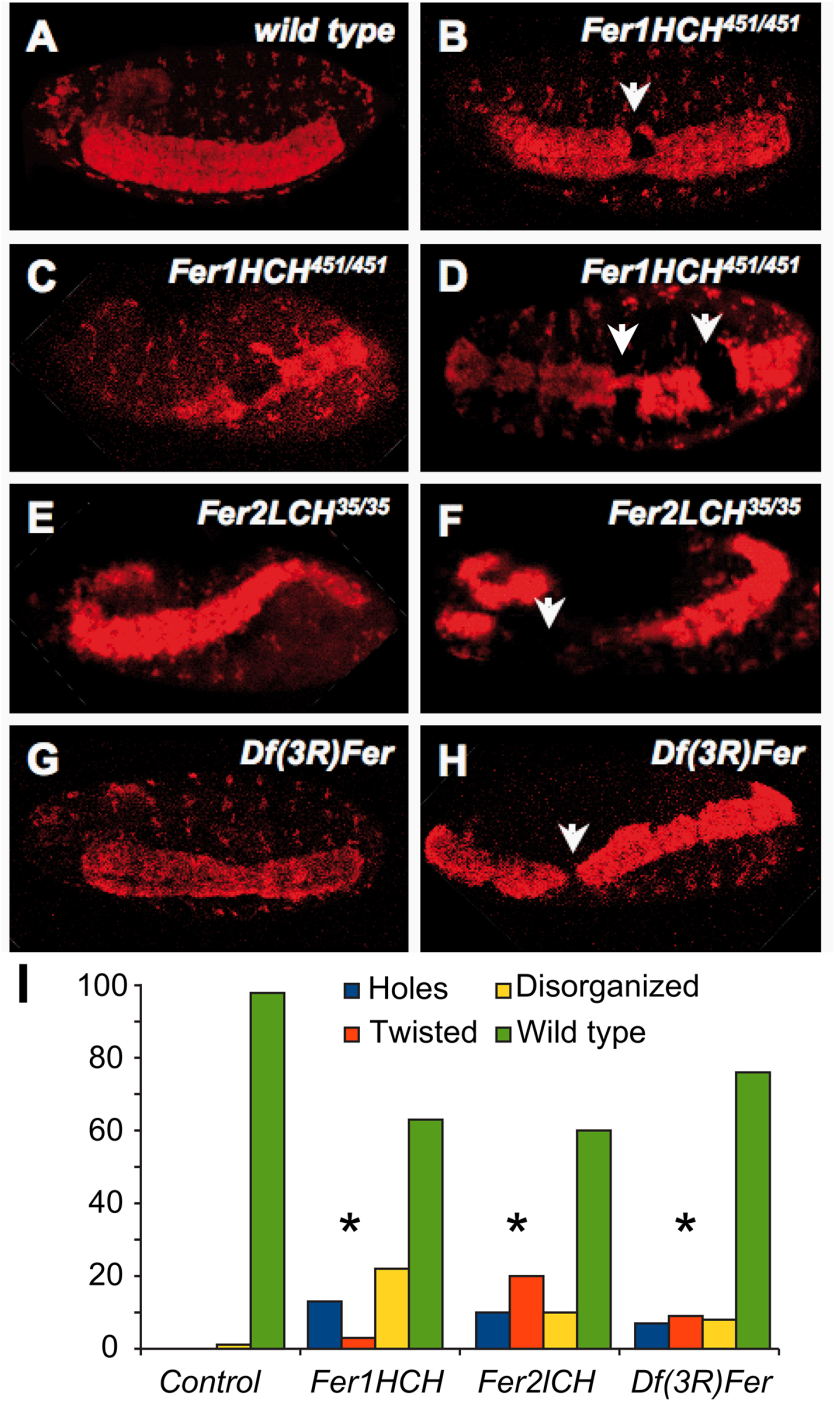
Ferritin mutants result in CNS phenotypes, as revealed by ɑ-Elav staining. (A) Mutant CNS (B-H) appear twisted and irregular (E, G); often, holes are seen within the ventral nerve cord (white arrows). Holes can range in sizes from small, partially (B, C) or completely (H) interrupting the ventral nerve cord, to large (F). There can also be multiple holes (D), as compared to wild type. Embryonic brains are also disrupted (F, opposite white arrow). Embryos are photographed at stages 14–15. (I) Quantification and distribution of CNS defects in different ferritin mutants that present CNS defects; statistical difference compared to control following a Chi squared test, at p<0.0001, and is denoted by an asterisk (n = 32, 10, and 138 embryos, respectively).

**Fig 4:**
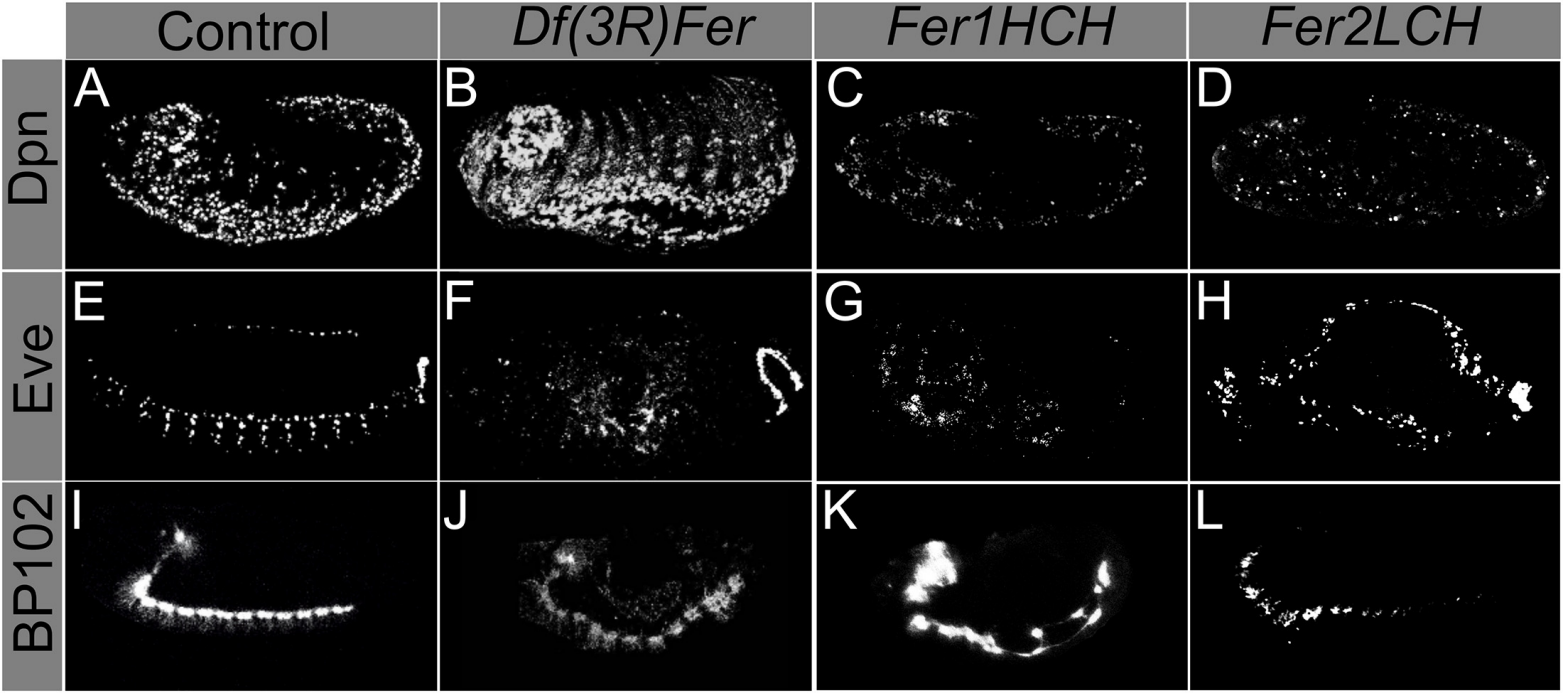
Neuroblast and ganglion mother cells populations and neuronal axons derived from embryonic neurons are affected in ferritin mutants. Neuroblast populations, as marked by Dpn (A, control) are affected in ferritin mutants (B-D). Ganglion mother cells (E, control) are affected in ferritin mutants (F-H). Axons emanating from the brain and ventral nerve cord have a stereotyped pattern in normal development (I). In ferritin mutants, the axons form but are disorganized (J-L).

We then stained embryos with antibodies against several neuronal markers. Anti-Elav, which marks neuronal nuclei, showed that some ferritin mutants harbor holes in the abdominal CNS segments (Figs 2 and 3). More severe phenotypes were also present, albeit in fewer embryos, including aberrant condensation of the CNS, twisted CNS, and loss of parts of the brain and peripheral nervous system (PNS). Importantly, and consistent with our analysis of the cuticle phenotypes discussed above, these phenotypes were observed with both ferritin alleles and with the 2.2 kb genomic deletion that specifically deletes both *Fer1HCH* and *Fer2LCH* (Fig 3B-3H, distribution of defects in 3I, compared with the control 3A). These phenotypes were significantly different from control embryos (Fig 3I). The developing CNS consists of at least four types of cells: neuroblasts, ganglion mother cells, neurons, and glia. Neuroblasts are CNS precursor cells and give rise to ganglion mother cells; and these, in turn, give rise to neurons and glia [36]. In order to test whether neuroblasts and ganglion mother cells were affected in ferritin mutant embryos, we performed antibody staining against Deadpan (Dpn) and Evenskipped (Eve) [37,38]. Anti-Deadpan staining, which marks all neuroblasts, shows that CNS defects are already present within neuroblast cell lineages in at least some mutant embryos (Fig 4A-4D). As expected for early CNS defects, mutant embryos can also be found where Eve positive ganglion mother cells are affected (Fig 3E-3H). CNS defects are detected from the time neuroblast cell lineages are specified in some mutant embryos, at stages 7–8, precisely during germband extension-retraction, when the earliest cuticle defects are seen. These early defects may result in more maturecontorted and aberrant nervous systems, as seen with anti-Elav.

In order to examine other aspects of later embryonic CNS stages (besides anti-Elav), we used anti- BP102 antibody to study condensed CNS and axonal trajectories in ferritin mutant embryos. Anti-BP102 stains axons and CNS. We studied the condensed ventral nerve cord and axons. Anti-BP102 staining revealed that in some mutant embryos the CNS ventral nerve cord is aberrant and contorted, and that axons are misguided (Fig 3I-3L). Altogether, CNS defects occur throughout CNS development.

### Ectopic apoptotic activation in ferritin mutants

**Fig 5:**
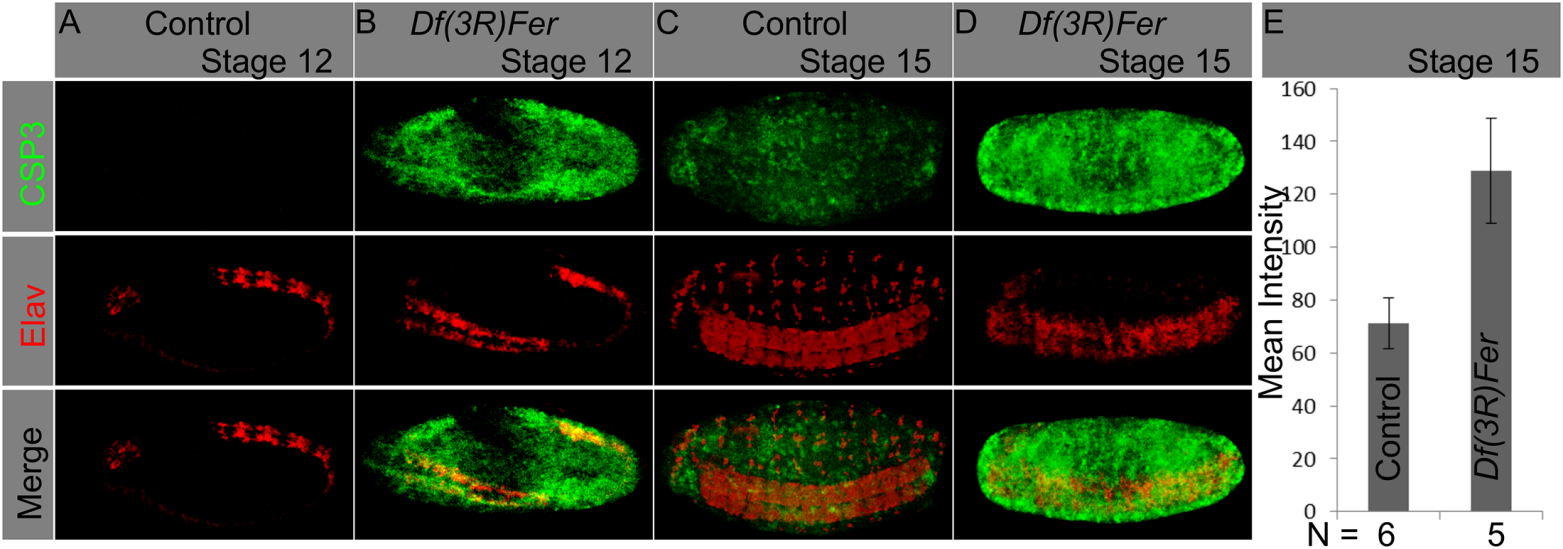
Ferritin mutants cause apoptosis in the CNS and other tissues. (A-D) Whole embryos were stained with an α-CSP3act marking apoptotic cells (green), and an α-Elav marking neurons (red). Ectopic apoptosis was observed in ferritin mutants from stage 12 onwards; at this stage it was mostly restricted to the neurogenic region (B). At stage 15 apoptosis covers most mutant embryonic tissues (D). Quantification of the mean intensity value on CSP3act staining in control and ferritin mutants at stage 15 show a significant difference, with higher levels of staining in mutant embryos (n = 5; T-test, p = 0.0113).

What are the consequences of ferritin loss in affected tissues? There are links between iron metabolism and apoptosis [39–44]. We hypothesized that in the mutants; disrupted CNS could lead to cell death by an apoptotic mechanism. To test this hypothesis we used an antibody that recognizes solely the cleaved, activated caspase3 in cells as an apoptosis marker [45]. In contrast to control embryos at stage 12 where no apoptotic signal was detected (Fig 5A), ectopic apoptotic activation appeared in mutant embryos (Fig 5B). By stage 15 of embryogenesis control embryos have a weak and restricted apoptotic signal (Fig 5C), whereas in the mutant embryos this signal was massive and covered most of the embryo (Fig 5D; significantly different from control, Fig 5E). Similar patterns were seen with *Fer1HCH451* and *Fer2LCH35* homozygous mutant embryos (S3 Fig). Thus, early apoptotic activation in ferritin mutants after we see CNS anatomical defects, and subsequent generalized apoptosis, suggest that ferritin mutants may suffer apoptosis as a direct consequence of lack of the ferritin complex (again both mutant alleles show a qualitatively similar apoptosis phenotype), ultimately affecting many tissues.

### The *Drosophila* DMT1 homolog, *Malvolio*, is upregulated in ferritin mutants

Mutations in *Mvl* result in reduced iron within intestinal iron storage cells, but also in the whole body [46]. *Mvl* mutants can suppress both intestinal iron accumulation resulting from ferritin [26] and from Multicopper Oxidase-1 (MCO1) misregulation [47]. A recent paper by the same group shows that the likely reductase for this enzyme is ascorbate; its influence on iron homeostasis is conserved in mosquitoes [48]. In view of the above findings, we used *Mvl97f*, a P-element insertion mutant carrying a *LacZ* reporter for gene activity that also leads to reduced *Mvl* expression [24,46] to ask whether *Mvl* and ferritin genes interact.

**Fig 6:**
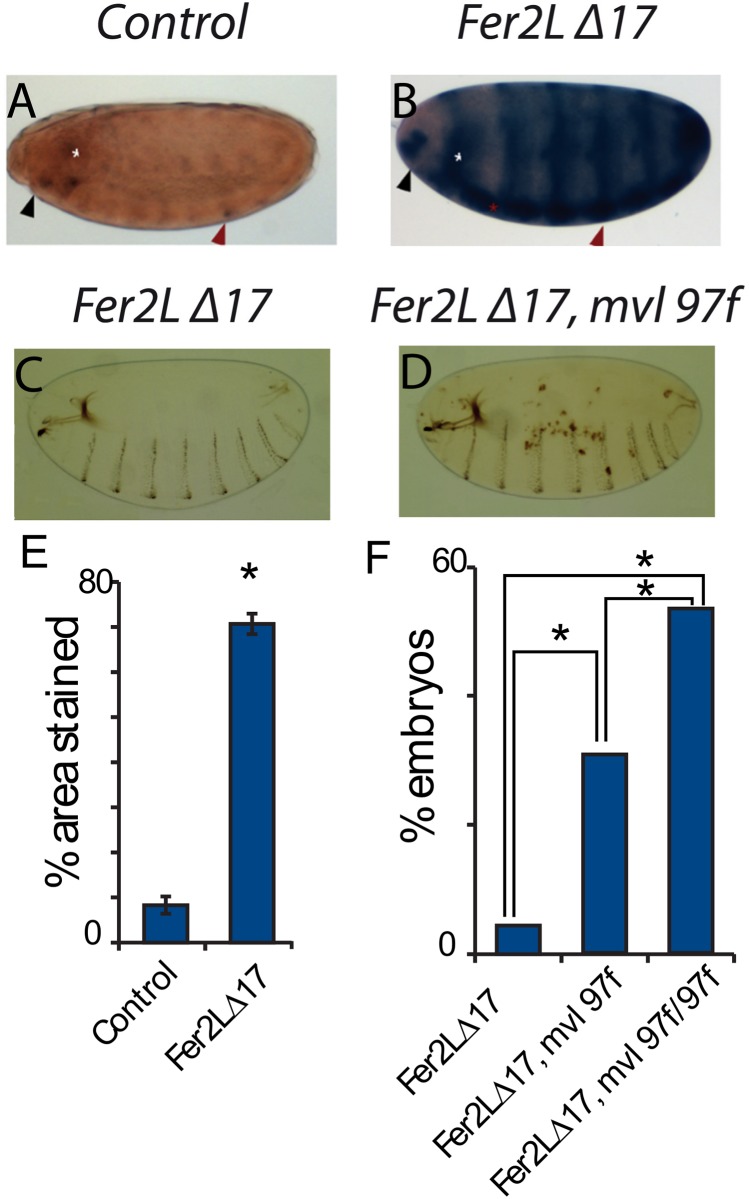
Ferritin genes interact genetically with the DMT1 homolog *Mvl*. (A) The *Mvl^97f^-LacZ* line shows a spatially restricted expression pattern for *Mvl*, mainly in the head region, the brain, and a segmentally repeated pattern. (B) In a ferritin homozygous mutant background, *Mvl^97f^-LacZ* expression increases. Black arrows denote the head region, white asterisk the embryonic brain, red asterisk the ventral nerve cord, and red arrows mark the segmented expression pattern. Introduction of a *Mvl^97f^* allele into a ferritin mutant background resulted in the appearance of necrotic patches in the cuticle (C, D). (E) Quantification of the total area covered by LacZ staining in control and ferritin mutant backgrounds, (p<0.0001; T-test, asterisk). (F) Quantification of the number of embryos showing necrotic patches with one or two *Mvl^97f^* alleles; statistical difference using a Chi squared test with p<0.0001 is shown by an asterisk.

In control embryos, LacZ showed a very restricted pattern of *Mvl* expression consistent with previous studies [21,24]. However, in a mutant ferritin background, *Mvl*- driven LacZ is upregulated, its levels increasing as development proceeds (Fig 6A, 6B and 6E). We hypothesize than in the absence of functional ferritin, iron depleted cells upregulate *Mvl* in an effort to curve iron depletion. Quantitation showed a significant difference of expression (Fig 6E). Significantly, the addition of one copy of the *Mvl97f* hypomorphic allele into a ferritin-depleted embryo resulted significantly in the appearance of necrotic patches, which were rarely present in *Mvl97f* or in ferritin homozygous mutants alone (Fig 6C, 6D and 6F).

### Ferritin expression, localization and trafficking during development

Ferritin might participate in an iron import pathway [26]. The above interaction between DMT1 and ferritin is reminiscent of recent findings in mammals. Indeed, the mammalian ferritin receptor SCARA5 is upregulated in the absence of the transferrin receptor [10].

In order to study this proposed transport function of ferritin, we used the *Fer1HCHG188* mutant allele. *Fer1HCHG188* is a mutant *Fer1HCH* allele, shown to generate a chimeric GFP-Fer1HCH protein that faithfully mimics the endogenous Fer1HCH pattern in heterozygous condition [17,32,35,49]. The GFP tag in homozygosis is thought to block the correct function of ferritin, because in homozygotes all the H-subunits carry a GFP tag, and embryonic development fails [17]. *Fer1HCHG188/+* flies are viable as heterozygotes, showing a dominant mild effect: a small reduction in iron accumulation within ferritin [34].

**Fig 7:**
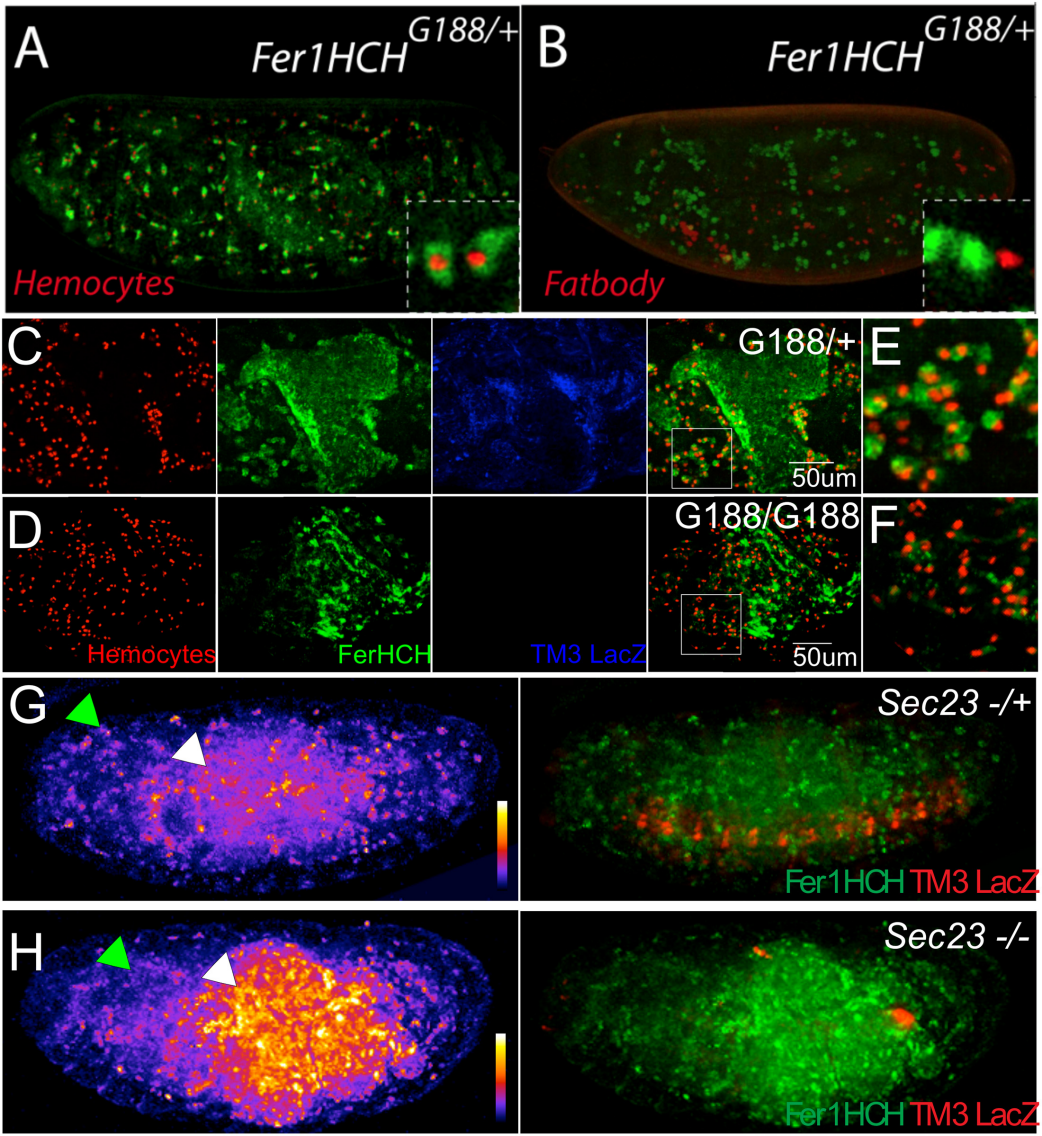
Marked ferritin accumulation in embryos with different genetic backgrounds. *Fer1HCH* protein was visualized in all embryos using the *Fer1HCH^G188^* GFP trap line (green). In stage 17 *Fer1HCH^G188/+^* embryos that successfully complete development, ferritin accumulates mainly in the midgut and hemocytes. (A) The GFP-Fer1HCH signal is found in hemocytes marked by *cg* driving nuclear RFP, but not (B) in the fat bodies marked by the fat body driver FB driving nuclear RFP. (C-F) *Fer1HCH^G188^* homozygous embryos, which die like other ferritin mutants, also show intestinal ectopically localized ferritin accumulation (D) and much reduced hemocyte ferritin accumulation (F), compared to heterozygous controls (C, E). (G, H) Blocking the secretory pathway using a homozygous mutant *sec23^j13C8^* background leads to a decrease of ferritin levels in the hemocytes (green arrowheads),; and ectopic accumulation in the intestine (white arrowheads). Intensity of GFP::Fer1HCH flurescence is shown in the left side of panels G and H using a fire scale. The fire scales are shown in the lower right side of each, with warmer colors (red-orange-yellow-white) denoting higher intensity of fluorescence, and colder colors (purple-blue) denoting lower levels of fluorescence for the green channel. Note higher fluorescence levels in the *sec23* homozygous mutant embryo (H). A merged image showing GFP::Fer1HCH fluorescence and control RFP staining (G) due to the balancer chromosome is shown to the right side of each panel.

During stages 16–17 of embryonic development GFP tagged Fer1HCH is present in hemocytes in *Fer1HCHG188* heterozygotes (Fig 7A). Hemocytes are large cells that are loosely associated with peripheral tissues circulating in the hemolymph, where they function as both phagocytic and immune cells [50]. There is no report of ferritin mRNA expression in hemocytes. In order to confirm that the large, ferritin-accumulating cells were actually hemocytes we used the *Cg-Gal4* line to drive expression, exclusively in hemocytes, of a nuclearRFP in *Fer1HCHG188/+* embryos. GFP-Fer1HCH is present in the same cells as Cg-nRFP (Fig 7A).

Ferritin mRNAs have been located in the fat body [17]. In a similar experiment as above, we used *Fer1HCHG188/+* and tagged the fat body with FB-nRFP, a fat body specific Gal4 line. *Fer1HCHG188* GFP and FB-nRFP are not coincidental (Fig 7B). If the ferritin mRNAs are translated in the fat body, they are either degraded quickly or transported elsewhere. A likely target candidate for transport is the hemocytes, where conversely there are no ferritin mRNAs; rather ferritin protein is present.

We noticed that homozygous mutant *Fer1HCHG188* embryos showed a clumped and concentrated accumulation of GFP-Fer1HCH than similarly staged heterozygous *Fer1HCHG188* embryos (Fig 7C and 7D). Homozygous *Fer1HCHG188* embryos have a lower and restricted GFP-Fer1HCH expression; this is mainly seen in the intestinal region (Fig 7D), suggesting either degradation of the mutant protein or that mutant, non-functional heteropolymers (composed exclusively of GFP- Ferritin1HCH and Ferritin 2LCH subunits), are not trafficked. The intestine could also be a possible source of transported ferritin, as iron from the diet is accumulated there and both ferritins mRNAs and proteins are present there (Fig 7C). Fewer hemocytes have *Fer1HCHG188 –*derived GFP in the mutant (Fig 7E and 7F), again consistent with ferritin transport from intestinal and / or fat body sources.

To test further whether ferritin is delivered from other embryonic tissues, we blocked the intracellular secretory pathway by means of a lethal mutation in Sec23. s*ec23j13C 8*is a P-element insertion in the 5’ UTR of *sec23*, expected to eliminate or severely attenuate gene function [51]. If ferritin is indeed transported during embryogenesis, blocking the secretory pathway will impede its exit from the cells where it is originally transcribed. Ferritin was detected clumped in *sec23j13C8/j13C8* mutants (compare Fig 7H to 7G); GFP-Fer1HCH aggregates were detected mainly around the midgut (Fig 7H) in a similar expression pattern as that observed in homozygous *Fer1HCHG188* mutants. This is consistent with ferritin trafficking during embryonic development.

Thus, in embryos impaired in the secretory pathway some cells fail to accumulate ferritin, suggesting that in wild type embryos ferritin may be actively secreted into the hemolymph. We then made use of two constructs, UAS-mcherry::Fer2LCH and UAS-GFP::Fer1HCH (Gambis, Steller & Mollereau, personal communication), and expressed them using the hemocyte *Cg-Gal4* line. We found that embryonic expression of the ectopic, tagged ferritin subunits was largely, although not absolutely, coincidental, and that expression was seen in cells other than hemocytes (S4 Fig) in late embryos.

We also used the same fluorescently tagged ferritin constructs to see whether these tagged ferritin proteins could also be expressed and transported in larvae. We used *Fer2LCH-Gal4* to drive expression (S5A and S5B Fig). We found expression of both constructs in the midgut, as expected (due to *Fer2LCH-Gal4*), but also in Garland cells, where no *Fer2LCH-Gal4* expression is seen, implying ferritin transport. We also used two other drivers: *elav-Gal4* for expression in neurons (S5C Fig) and *repo-Gal4* for expression in glial cells (S5D Fig). In both cases, fluorescence is also seen in Garland cells, and again, implying ferritin transport. Garland cells are nephrocytes, part of the larval fly hemolymph filtration system [52], and may come in contact and accumulate ferritin circulating in the hemolymph.

Taken together, all these data point that ferritin traffics between tissues, and that hemocytes and hemolymph may play key roles in this trafficking process, conveying ferritin from one tissue to another. Such a role for hemocytes has been suggested in the context of tissue communication in the innate immune response [53].

## Discussion

Insect embryos must course through development with limited amounts of iron, provided during oogenesis by the mother, in part *via* ferritin. It seems reasonable to assume that tissues developing at different rates present different iron requirements, and therefore iron transport must be of vital importance for normal development. This may also mean that ferritin is used for trafficking from storage sites, as development proceeds, to tissues and cells where it is required, as an iron conveyor.

Here we show that ferritin mutants die during embryogenesis with a wide range of phenotypes. Ferritin mutants first show cuticular defects after gastrulation, during germband extension-retraction and the formation of the nervous system, if we classify lack of cuticle formation only as a late occurring defect. Around stage 12, at about the initiation of dorsal closure, there is already ectopic apoptosis, and it becomes more prevalent and intense as embryonic development continues. Judging from the generalization of apoptosis, not all portions of the embryo are affected in a similar manner, as ectopic apoptotic signal is first seen in the neurogenic region, before becoming generalized. Most zygotic mutants do from cuticle, implying that even at a stage where there is prevalent apoptosis, some cells survive (differentiating epidermis cells) enough to secrete cuticle at the end of embryogenesis. In contrast, the CNS is affected from the start.

If iron provided maternally by ferritin is reduced or completely missing, the same phenotypes are present, but particularly, embryos not forming cuticle become prevalent. This implies that the ‘no cuticle’ phenotype stems from a very early ferritin requirement manifested late by lack of cuticle deposition. Ferritin damage ends in ectopic apoptosis, eventually generalized throughout the embryo.

We also provide evidence of ferritin trafficking. Blocking the secretory pathway during development causes abnormal ferritin distribution, suggesting an additional iron-trafficking role for ferritin. Our ectopic studies point in the same way, at least for the ectopically produced ferritin subunits. This may be responsible for differences in ferritin mRNA and protein expression. Ferritin mutations also cause an up-regulation of *Mvl*, probably as a means to stimulate iron uptake from iron deprived cells.

Tang and Zhou demonstrated the importance of ferritin expression in the larval intestine [26]. Our studies in larval intestine are consistent with this, and suggest that ferritin expression in the gut is a starting point for ferritin larval function.

Our results also point to both ferritin subunits being jointly required in the embryonic CNS. In adult flies, in contrast, both genes might also have different functions. RNA interference in subsets of neurons against *Fer2LCH* but not against *Fer1HCH* disrupted circadian rhythms[54]. Furthermore, some cell types, including commonly used cell culture lines [55,56], only express *Fer1HCH* and not *Fer2LCH*. Overexpression of either *Fer1HCH* or *Fer2LCH*, or both subunits simultaneously in *Drosophila* glia [43] or neurons [53,57] resulted in qualitatively different responses. We also find that embryonic ectopic expression of both ferritin subunits leads to some cells differentially expressing one of the two genes. Use of the *elav-Gal4* driver to silence either *Fer1HCH* or *Fer2LCH* resulted in viable adults with perturbed circadian behavior [54] and apparent neurodegeneration [26]. RNA interference is known to cause reduced expression but not complete silencing of its targets, which may explain why ferritin RNAi flies survived to adulthood. In addition and consistent with our early CNS defects results, overexpression of ferritin subunits in this last study with *elav-Gal4* failed to rescue their respective mutants [26], implying an early ferritin neuronal requirement, before the *elav-Gal4* transgene is active, as seen in our staining experiments with early CNS markers. Furthermore, disrupting ferritin levels (either by reductions or ectopic expression may provoke disequilibrium of ferritin proteins and explain the differing results observed.

We conclude that both ferritins are normally required, but that sometimes regulation in vivo is effected primarily via Fer2LCH. Thus, the question of how different cell types regulate in vivo the two ferritin genes and subunits and whether they always act in concert requires further investigation.

## Materials and Methods

### Fly stocks

All flies were *Drosophila melanogaster* Meigen. As a control strain *y*, *w* flies were used. *Fer1HCH451* and *Fer2LCH35* are P(ry[+t7.2] = PZ) insertion alleles generated during a large-scale mutagenesis screen [58], and have been partially characterized elsewhere [17]. They were obtained from the Bloomington Drosophila Stock Center (BDSC); stock numbers #11497 and #11483, respectively. *Fer2LCHΔ17* was generated from an imprecise excision of *Fer2LCHEP1059* (described in Flybase) and interferes with expression of both genes, as confirmed by complementation crosses. *Df(3R)Fer* was a gift from Alexis Gambis, Bertrand Mollereau, and Hermann Steller and is a 2.2 kb deletion disrupting specifically *Fer1HCH* and *Fer2LCH* [34]. To generate germline clones, *Fer1HCH451* and *Fer2LCH35* were recombined unto FRT82 containing chromosomes [59]. *Fer1HCHG188* is a protein trap line and has been extensively described elsewhere [17,60]. *Mvl97f* is an homozygous viable *P(lacW)* insertion obtained from the (BDSC stock #5151) (Rodriguez, et al. 1995). *Sec23j13c8* mutant is a *P(lacW)* insertion within the 5'UTR of *Sec23* [51]; BDSC stock #10218. *Cg-Gal4* (BDSC stock #7011) was used to drive expression in the hemocytes, *drm-Gal4* (BDSC stock #7098) in embryonic gut and scattered cells around the epidermis, *FB-Gal4* in the fat body (P{GAL4}fat; Flybase ID; FBti0013267) [16,61,62]. UAS-mcherry::Fer2LCH and UAS-GFP::Fer1HCH were a gift from Alexis Gambis, Hermann Steller and Bertrand Mollereau (Gambis, Steller, and Mollereau, personal communication), and will be described elsewhere. In cases where recombinant or double balanced stocks were needed they were generated following conventional crossing schemes.

### Iron diets

Flies were raised for 3 successive generations on standard medium supplemented with 200 μM Bathophenanthrolinedisulfonic acid disodium salt (SIGMA #B1375) referred to as BPS in the text or with 1 mM ammonium iron (III) citrate (SIGMA #F5859) referred to as FAC. Adults were used for embryo collections. Protein extracts from female adults were also analyzed by non-reducing SDS- PAGE, confirming the differential accumulation of ferritin in flies raised on the respective diets.

### Immunohistochemistry and confocal imaging

Following dechorionation with a commercial bleach solution, embryos from overnight collections were devitellinized and fixed in a 1:1 mixture of heptane and 36% formaldehyde for 5 minutes and then washed in methanol. Embryos were then stored at -20 C or rehydrated, and used for staining. Primary antibodies used were: rat α-Elav 1:100, mouse α-BP102 1:100, mouse α-Eve 1:100 (Developmental Studies Hybridoma Bank, Indiana, USA); α-activated Caspase 3 1:100 (Cell Signaling, USA); and rat α-Deadpan 1:2, a gift from Cheng-Yu Lee. Secondary antibodies used were: Alexa flour 546 α-rat 1:100 (Santa Cruz Biotechnology, USA), Cy5 α-mouse 1:1000, Cy3 α-mouse 1:1000, FITC α-rabbit 1:1000 (Zymax, USA). Signal from α-deadpan staining was increased with the ABC kit from Vectastain (USA). A 510 Meta and 780 Duo confocal microscopes (Zeis, Germany) were used for fluorescent imaging, and images were processed with Zeiss software and ImageJ. Homozygous mutant embryos were selected by lack of TM3GFP of TM3LacZ. Accumulation of GFP::Fer1HCH in *Sec23* mutants was evaluated using the fire LUT of ImageJ.

### Cuticle preparations and X-Gal staining

Embryos were collected in agar containing plates for 12 hours and incubated for another 36 hours at25°C. Viable first instar larvae were removed from cultures. The cuticles of unhatched (dead) embryos were dechorionated and mounted in Hoyer's medium and incubated for 24 hours at 50°C to digest soft tissues. Resulting cuticles were then viewed and photographed with dark field optics in a compound microscope (Nikon, Japan). For X-Gal staining embryos were fixed and stained with X-Gal using standard procedures. Both controls and experimental embryos were incubated in parallel for the same amount of time to allow for direct comparisons.

## Supporting Information

S1 Fig Ferritin expression can be modulated by iron availability. Ferritin complexes were revealed by Coomasie staining and identified by size, when either flies were fed Bathophenanthroline Sulfate (BPS) to reduce iron availability (BPS is an iron chelator), or fed extra iron (FAC).
(TIF)

S2 Fig *Fer1HCH^451^* is a functional ferritin enhancer trap line. β-Galactosidase expression is normally restricted to the iron region in the larval midgut, but expression is enhanced in the anterior midgut (AMG) when iron fed, as occurs in wild type larve (Mehta et al. 2009).
(TIF)

S3 Fig Apoptosis in ferritin mutants. Apoptosis revealed with an α-active caspase 3 antibody (CSP3act) marking apoptotic cells (green), and an α-Elav marking neurons (red) in *Fer1HCH^451/451^* (A) and *Fer2LCH^35/35^* (B) mutant embryos.
(TIF)

S4 Fig Embryonic ectopic expression of fluorescence-tagged ferritin subunits reveals embryonic dynamic ferritin transport of tagged ferritins. (A) Control embryo where embryonic hemocytes are revealed by the *Cg-Gal4* line and UAS-RFP (red fluorescent protein). *Cg-Gal4* drives RFP expression specifically in hemocytes. (B-D) Overexpression of both ferritin tagged subunits in the hemocytes results in ferritin accumulation in these cells (asterisk), but also in other tissues (arrowhead). UAS-mcherry::Fer2LCH (UAS-cherry:Fer2LCH) tagged ferritin expression is in (B), and UAS-GFP::Fer1HCH (UAS-GFP:Fer1HCH) is in (C). A merged image in shown in D. The white arrowhead points to embryonic tissues harboring fluorescence other than hemocytes. The asterisk marks hemocytes. (E-G) Higher magnification images of a similar embryo revealing partial co-localization of tagged ferritin subunits driven by *Cg-Gal4*, where cells accumulating one subunit but not the other are seen (dashed circles). UAS-mcherry::Fer2LCH expression is shown in (E), UAS-GFP::Fer1HCH is shown in (F), and a merged image is shown in G. The tagged ferritin lines will be described elsewhere (Gambis, Steller, and Mollereau, personal communication).
(TIF)

S5 Fig Ectopic expression of tagged ferritin subunits reveals dynamic ferritin transport in larvae. (A) Ectopic expression of RFP::Fer2LCH using a *Fer2LCH-Gal4* line results in ferritin accumulation in the anterior midgut and in Garland cells (dashed line). UAS-GFP was used as a transcription marker for *Fer2LCH-Gal4* activity (A’) and is detected in the anterior midgut but not in the Garland cells. Merged image in (A”). (B) Likewise, ectopic expression of GFP::Fer1HCH using *Fer2LCH-Gal4* also results in GFP::Fer1HCH accumulation in the anterior midgut and in Garland cells (dashed line). Nuclear RFP (B’) was used as a transcription marker for *Fer2LCH-Gal4* and is detected in the anterior midgut but not in the Garland cells. Merged image in (B”). (C) Ectopic expression of tagged ferritin subunits in neurons via *elav-Gal4* results in theiraccumulation in Garland cells, implying GFP::Fer1HCH (C) and RFP::Fer2LCH (C’) transport from neurons to Garland cells. Merged image in (C”) also shows localization of Garland cells around digestive tract (combined fluorescence and transmitted light image). (D) Glial expression via the *repo-*Gal4 driver of both tagged ferritin subunits (GFP::Fer1HCH (D) and RFP::Fer2LCH (D’) results in tagged their accumulation in a discrete portion of midgut enterocytes. Merged image in (D”).
(TIF)
