# Supplementary figures and images for: Ferritin Is Required in Multiple Tissues during Drosophila melanogaster Development
Source: PLoS One. 2015 Jul 20;10(7):e0133499. doi: 10.1371/journal.pone.0133499 (PMC4508113; doi:10.1371/journal.pone.0133499)

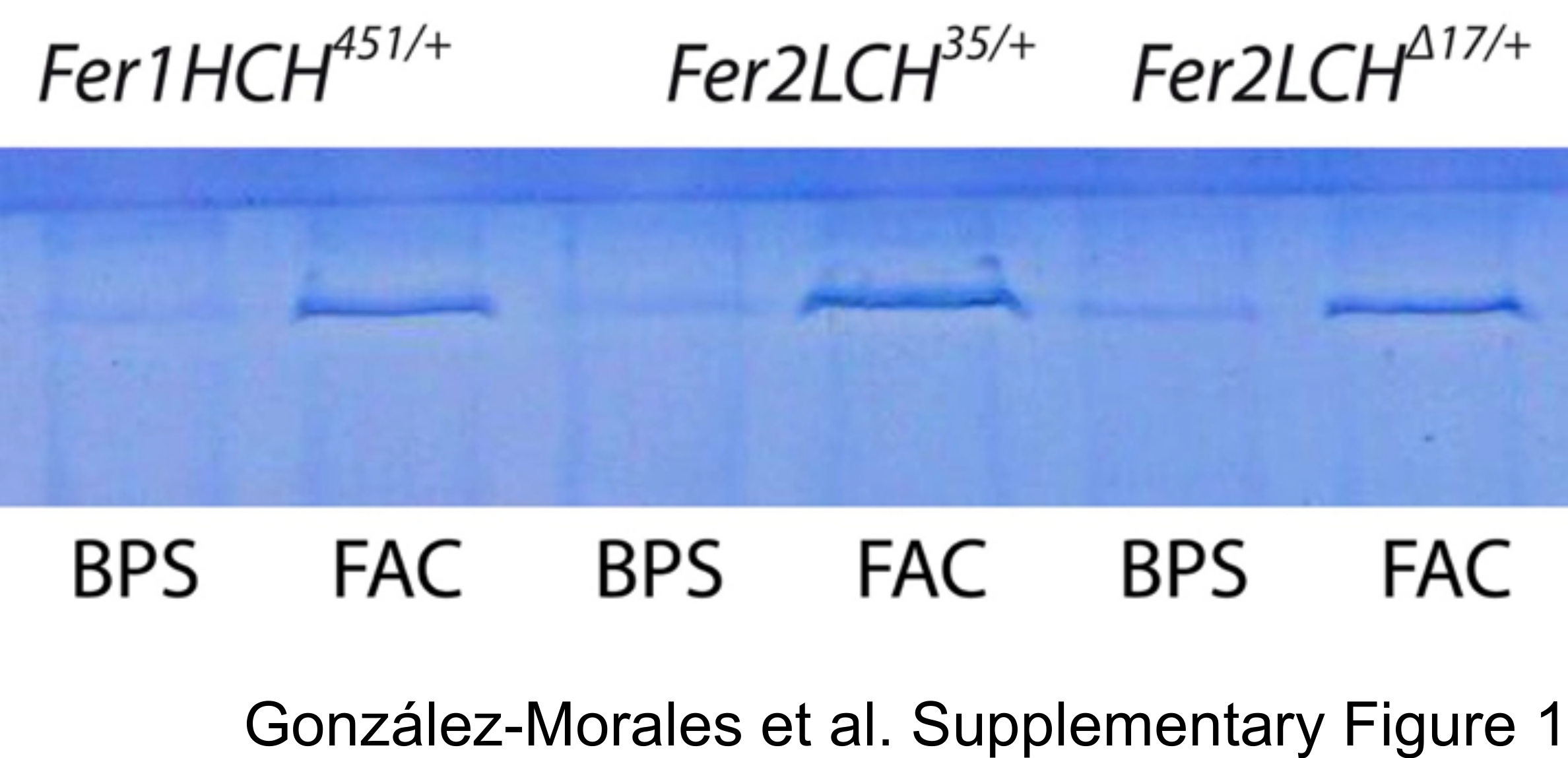

Supplement: S1 Fig — Ferritin complexes were revealed by Coomasie staining and identified by size, when either flies were fed Bathophenanthroline Sulfate (BPS) to reduce iron availability (BPS is an iron chelator), or fed extra iron (FAC). (TIF) [file pone.0133499.s001.tif]

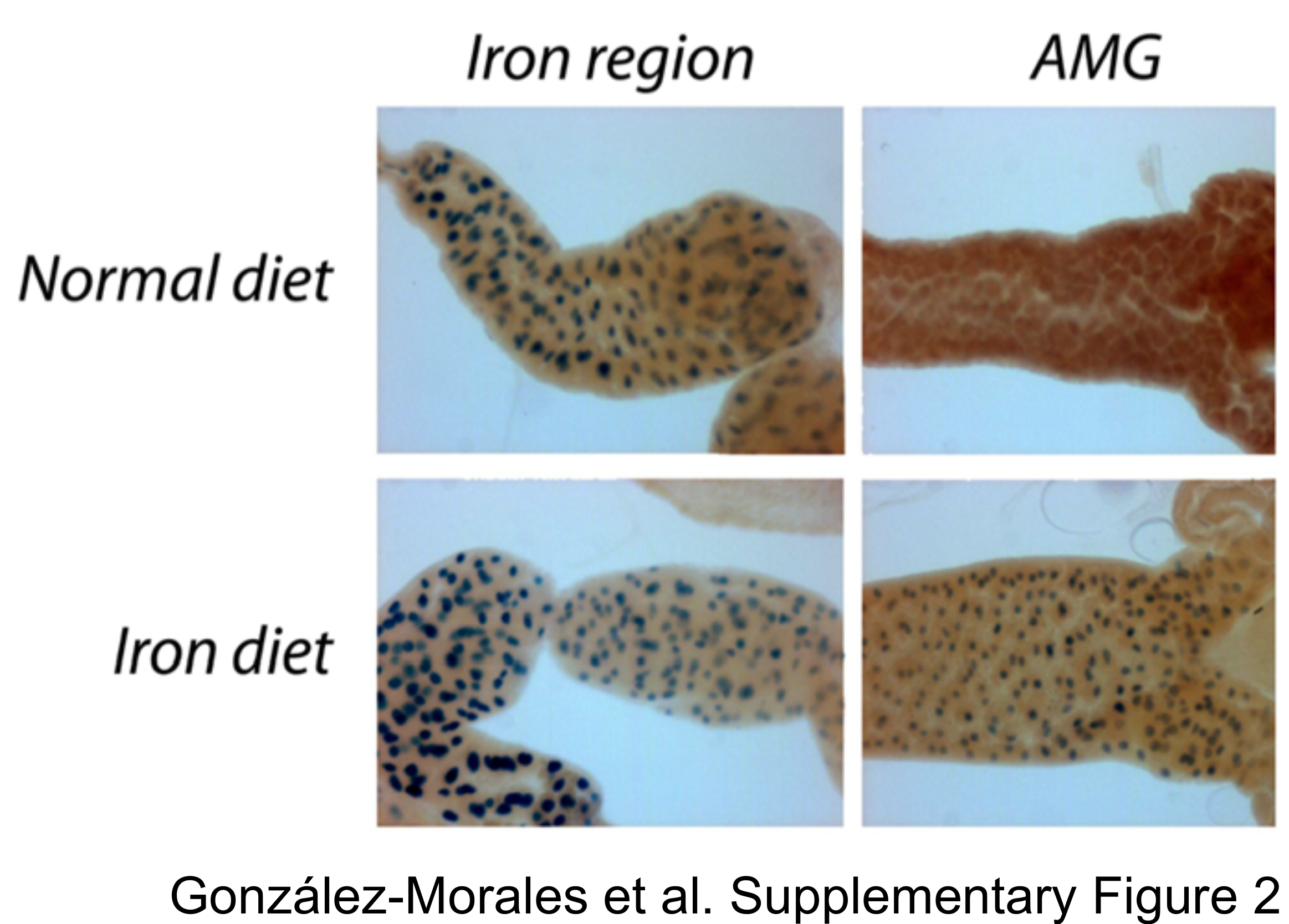

Supplement: S2 Fig — β-Galactosidase expression is normally restricted to the iron region in the larval midgut, but expression is enhanced in the anterior midgut (AMG) when iron fed, as occurs in wild type larve (Mehta et al. 2009). (TIF) [file pone.0133499.s002.tif]

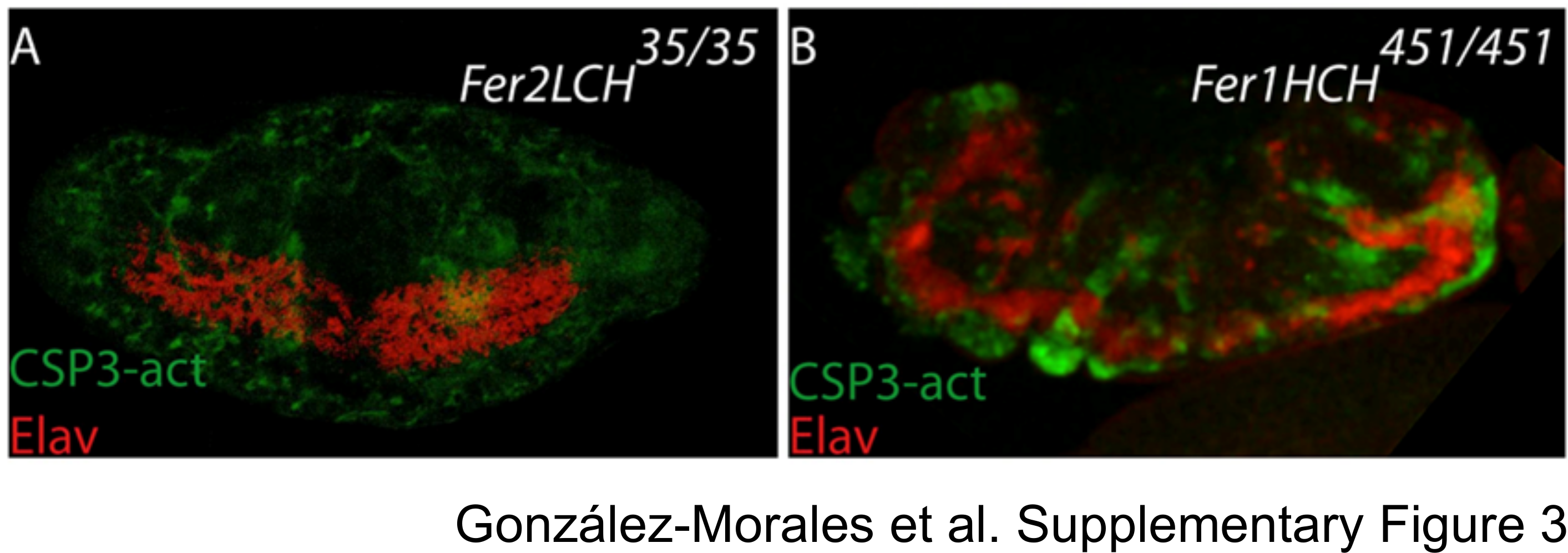

Supplement: S3 Fig — Apoptosis revealed with an α-active caspase 3 antibody (CSP3act) marking apoptotic cells (green), and an α-Elav marking neurons (red) in Fer1HCH451/451 (A) and Fer2LCH35/35 (B) mutant embryos. (TIF) [file pone.0133499.s003.tif]

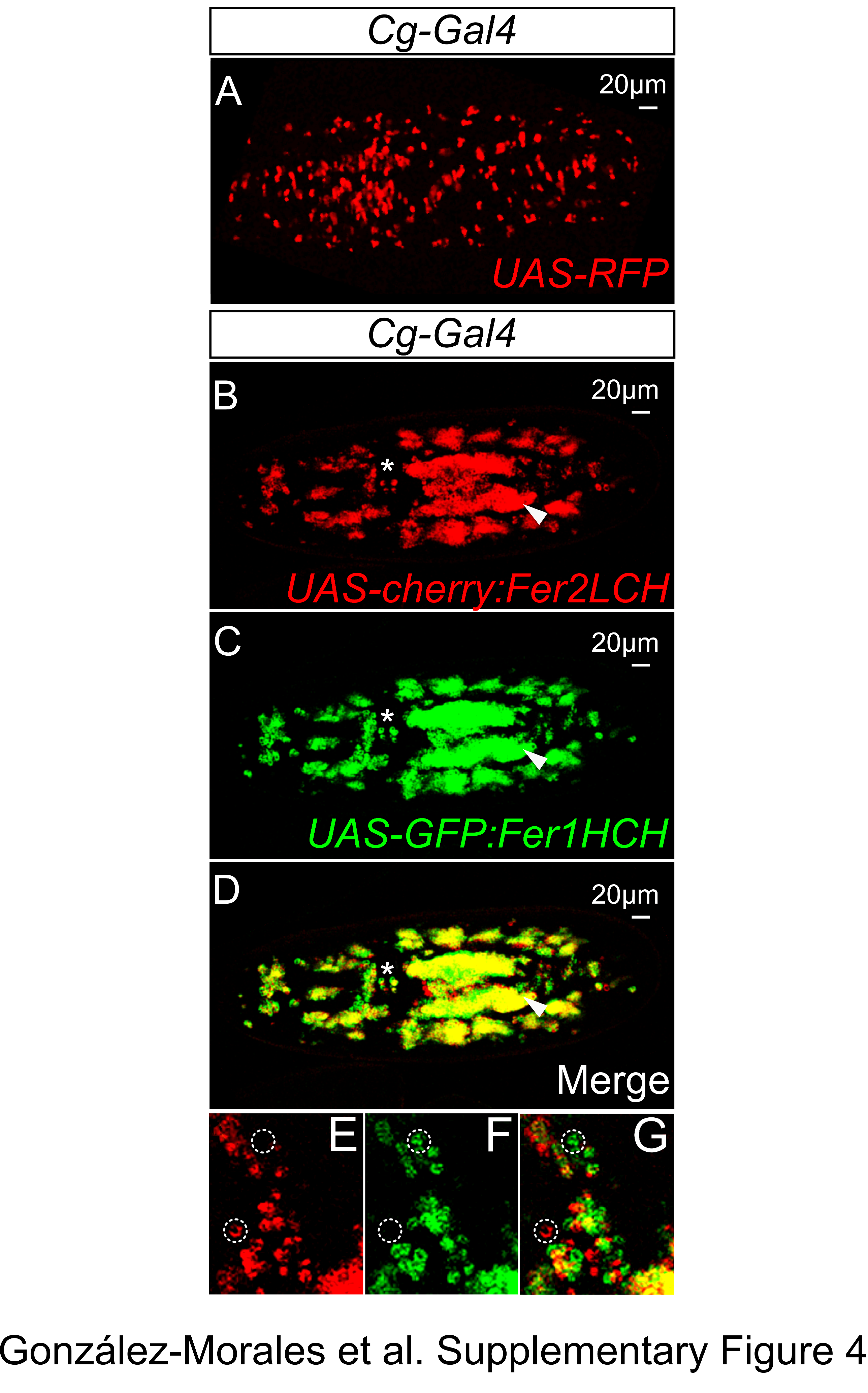

Supplement: S4 Fig — (A) Control embryo where embryonic hemocytes are revealed by the Cg-Gal4 line and UAS-RFP (red fluorescent protein). Cg-Gal4 drives RFP expression specifically in hemocytes. (B-D) Overexpression of both ferritin tagged subunits in the hemocytes results in ferritin accumulation in these cells (asterisk), but also in other tissues (arrowhead). UAS-mcherry::Fer2LCH (UAS-cherry:Fer2LCH) tagged ferritin expression is in (B), and UAS-GFP::Fer1HCH (UAS-GFP:Fer1HCH) is in (C). A merged image in shown in D. The white arrowhead points to embryonic tissues harboring fluorescence other than hemocytes. The asterisk marks hemocytes. (E-G) Higher magnification images of a similar embryo revealing partial co-localization of tagged ferritin subunits driven by Cg-Gal4, where cells accumulating one subunit but not the other are seen (dashed circles). UAS-mcherry::Fer2LCH expression is shown in (E), UAS-GFP::Fer1HCH is shown in (F), and a merged image is shown in G. The tagged ferritin lines will be described elsewhere (Gambis, Steller, and Mollereau, personal communication). (TIF) [file pone.0133499.s004.tif]

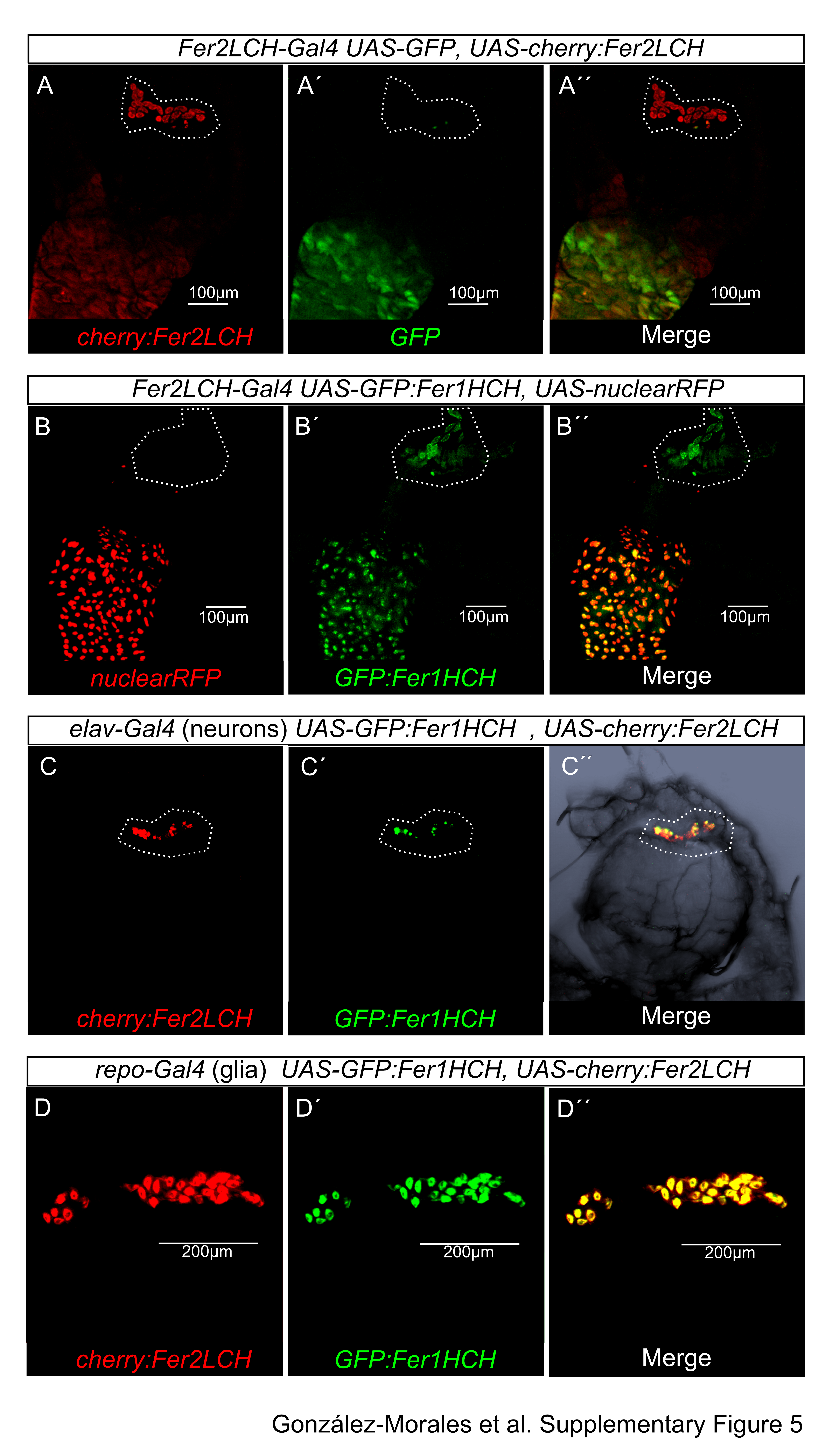

Supplement: S5 Fig — (A) Ectopic expression of RFP::Fer2LCH using a Fer2LCH-Gal4 line results in ferritin accumulation in the anterior midgut and in Garland cells (dashed line). UAS-GFP was used as a transcription marker for Fer2LCH-Gal4 activity (A’) and is detected in the anterior midgut but not in the Garland cells. Merged image in (A”). (B) Likewise, ectopic expression of GFP::Fer1HCH using Fer2LCH-Gal4 also results in GFP::Fer1HCH accumulation in the anterior midgut and in Garland cells (dashed line). Nuclear RFP (B’) was used as a transcription marker for Fer2LCH-Gal4 and is detected in the anterior midgut but not in the Garland cells. Merged image in (B”). (C) Ectopic expression of tagged ferritin subunits in neurons via elav-Gal4 results in theiraccumulation in Garland cells, implying GFP::Fer1HCH (C) and RFP::Fer2LCH (C’) transport from neurons to Garland cells. Merged image in (C”) also shows localization of Garland cells around digestive tract (combined fluorescence and transmitted light image). (D) Glial expression via the repo-Gal4 driver of both tagged ferritin subunits (GFP::Fer1HCH (D) and RFP::Fer2LCH (D’) results in tagged their accumulation in a discrete portion of midgut enterocytes. Merged image in (D”). (TIF) [file pone.0133499.s005.tif]
